# Cadmium-Induced Cell Homeostasis Impairment is Suppressed by the Tor1 Deficiency in Fission Yeast

**DOI:** 10.3390/ijms21217847

**Published:** 2020-10-22

**Authors:** Miroslava Požgajová, Alica Navrátilová, Eva Šebová, Marek Kovár, Miroslava Kačániová

**Affiliations:** 1 AgroBioTech Research Centre, Slovak University of Agriculture in Nitra, 949 76 Nitra, Slovakia; 2 Department of Genetics and Breeding Biology, Faculty of Agrobiology and Food Resources, Slovak University of Agriculture in Nitra, 94976 Nitra, Slovakia; alica.navratilova@uniag.sk; 3 Institute of Experimental Medicine, Czech Academy of Science, 14220 Prague, Czech Republic; eva.sebova@iem.cas.cz; 4 Department of Plant Physiology, Faculty of Agrobiology and Food Resources, Slovak University of Agriculture in Nitra, 94976 Nitra, Slovakia; marek.kovar@uniag.sk; 5 Department of Fruit Science, Viticulture and Enology, Faculty of Horticulture and Landscape Engineering, Slovak University of Agriculture in Nitra, 94976 Nitra, Slovakia; miroslava.kacaniova@gmail.com; 6 Department of Bioenergetics, Food Analysis and Microbiology, Institute of Food Technology and Nutrition, University of Rzeszow, 35-601 Rzeszow, Poland

**Keywords:** *Schizosccharomyces pombe*, Tor1, cadmium, ROS, morphology, ionome

## Abstract

Cadmium has no known physiological function in the body; however, its adverse effects are associated with cancer and many types of organ system damage. Although much has been shown about Cd toxicity, the underlying mechanisms of its responses to the organism remain unclear. In this study, the role of Tor1, a catalytic subunit of the target of rapamycin complex 2 (TORC2), in Cd-mediated effects on cell proliferation, the antioxidant system, morphology, and ionome balance was investigated in the eukaryotic model organism *Schizosaccharomyces pombe*. Surprisingly, spectrophotometric and biochemical analyses revealed that the growth rate conditions and antioxidant defense mechanisms are considerably better in cells lacking the Tor1 signaling. The malondialdehyde (MDA) content of Tor1-deficient cells upon Cd treatment represents approximately half of the wild-type content. The microscopic determination of the cell morphological parameters indicates the role for Tor1 in cell shape maintenance. The ion content, determined by inductively coupled plasma optical emission spectroscopy (ICP-OES), showed that the Cd uptake potency was markedly lower in Tor1-depleted compared to wild-type cells. Conclusively, we show that the cadmium-mediated cell impairments in the fission yeast significantly depend on the Tor1 signaling. Additionally, the data presented here suggest the yet-undefined role of Tor1 in the transport of ions.

## 1. Introduction

Cadmium (Cd) naturally occurs in ores together with zinc, copper, and lead, which makes volcanic activity the primary reason for a temporary cadmium boost in the environment. However, the relatively rare ion in the Earth’s crust represents nowadays a serious threat to human health, as its release due to anthropogenic activities has increased dramatically over the last few decades [1]. For industrial applications, cadmium is generated as a by-product during the production of non-ferrous metals from sulfide ores—in particular, zinc [2]. Cadmium is widely used in industry, mostly as an alloy component, as a PVC (polyvinyl chloride) stabilizer, for the corrosion-resistant electroplating of steel, for the production of nickel–cadmium batteries, and in the nuclear industry [3]. Due to the elevated industrial applications of cadmium, its annual global production has increased more than 1000 times since the beginning of the twentieth century. As the degradation of Cd is very limited, it persists in the ecosystem for long period and accumulates in crops, leading to its enhanced exposure to humans through the food chain. The human consumption of cadmium is estimated to reach 30 mg per day. Cadmium uptake followed by its accumulation in the organism is predominantly accomplished through a divalent metal transporter, however the molecular basis for this process remains unrevealed [4]. Cadmium, as a hazardous environmental pollutant, represents a potent toxicant to all forms of living organisms, including plants, animals, humans, bacteria, and fungi. Although the mechanisms of Cd toxicity and resistance vary among species, the fashion of its effects and the response of the organism largely depend on the form of the metal and the surrounding environment [5]. Cd-mediated enhancement of reactive oxygen species (ROS) production, its interaction with the DNA repair mechanism, and the induction of apoptosis impair cell proliferation, differentiation, and cell-fate decisions. Moreover, cadmium persists in the kidneys and liver, but it can be found in other tissues such as bone and placenta, and even at low concentrations is able to inhibit cellular respiration and oxidative phosphorylation, as it binds to the mitochondria [6].

The main regulator of cell growth and metabolism in response to nutrient availability and different environmental conditions is the serine/threonine target of rapamycin (TOR) kinase, which is structurally and functionally highly conserved from yeast to humans [7,8]. Rapamycin is a macrolide antibiotic initially isolated from *Streptomyces hygroscopicus* found on Easter Island, Rapa Nui, which gave it the name “rapamycin”. A single TOR kinase is found in mammalian cells (mTOR), while yeasts contain two TOR kinases, Tor1 and Tor2, of which the Tor2 kinase is essential for growth. In all the examined cell types, the TOR kinases serve as regulatory subunits of two functionally distinct multi-protein complexes—TOR complex 1 (TORC1) and TOR complex 2 (TORC2) [9]. The two complexes contain shared and complex-specific components and play crucial roles in the regulation of diverse signaling pathways responsible for the coordination of a range of fundamental cellular processes. Interestingly, only the activity of the TORC1 complex is associated with rapamycin sensitivity, and its regulation largely depends on nutrients [10]. TORC2 is resistant to rapamycin and was shown to regulate the cell wall integrity through the control of actin cytoskeletal reorganization and the regulation of actin cytoskeletal polarization [11]. Yeasts have long been used as a model system to study biological processes in higher eukaryotes. *Schizosaccharomyces pombe* (S. *pombe*, fission yeast) is a rod-shaped unicellular ascomycete which grows by polarized extension and divides through a central fission. Its genome comprises of only three chromosomes and was completely sequenced in 2002 [12], which was followed by extensive genetic analysis. Despite the inability of rapamycin to inhibit the cell growth or cell division of S. *pombe*, two TOR homologues were identified in the fission yeast, Tor1, a regulatory subunit of TORC2, and Tor2, a regulatory subunit of TORC1. As the nomenclature of the *S. pombe* TOR kinases is unfortunate, the *S. pombe* Tor1 is similar to *S. cerevisiae* Tor2, and the *S. pombe* Tor2 is similar to *S. cerevisiae* Tor1. Although the genetic similarity of the two S. pombe TOR homologs is 52%, their products play distinct roles in the organism. While Tor1 signaling is required under nutritional stress, extreme temperatures, and osmotic or oxidative stress conditions, the regulatory processes mediated through the Tor2 kinase are essential under normal growth conditions [13]. Hence, TOR-mediated signaling still represents a major focus of interest on one hand in terms of disease therapy, but on the other hand also in terms of the physiological dysregulation resulting from a contaminated environment [14,15].

In the presented study, we investigated what role the TORC2 catalytic unit, the Tor1 kinase, plays under cadmium-induced stress conditions in *S. pombe*.

## 2. Results

### 2.1. Cadmium Induces Cell Growth Alterations in A Dose Dependent Manner

**Figure 1:**
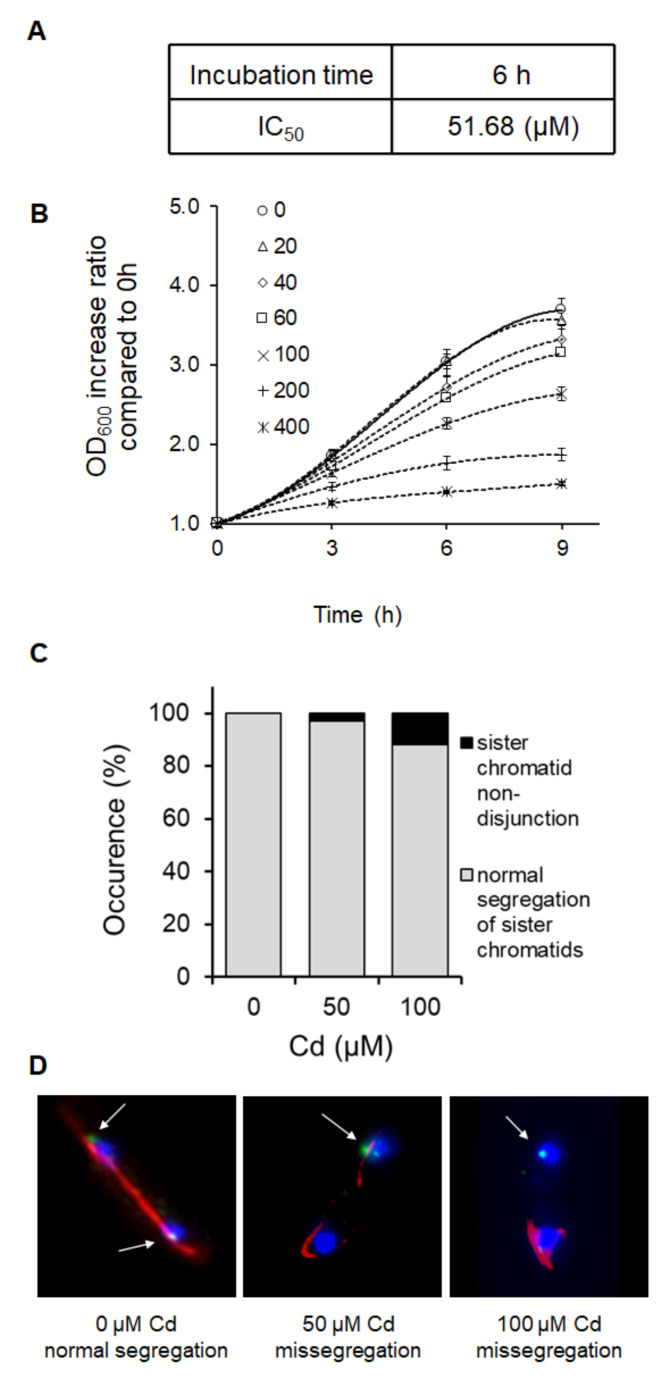
Cadmium affects chromosome segregation and impairs cell growth. (**A**) The half maximal inhibitory concentration (IC_50_) value represents the Cd concentration that reduces the growth of wild-type *S. pombe* cells to 50%. (**B**) Growth rate determination via optical density measurement at 600 nm (OD_600_) reveals the dose-dependent inhibition of the cell growth with increasing Cd concentrations. (**C**) Chromosome segregation in cells undergoing anaphase was determined with the use of the very useful Lac operator (lacO)/Lac repressor (LacI)-fused to the green fluorescence protein (GFP) system, which ensures the specific visualization of the second chromosome. Cd treatment enhances the occurrence of errors in the process of chromosome segregation. The graph represents the percentage of unsegregated sister chromatids of 100 counted cells. (**D**) Representative pictures of anaphase cells before and after the Cd treatment visualized by fluorescence microscopy. Red color represents mitotic spindle, blue is the nucleus, and the second chromosome is visualized as a green dot. White arrows indicate the position of the II chromosome, showing the normal chromosome segregation of the untreated control and non-disjunction of sister chromatids after Cd treatment.

*S. pombe*, a single-celled microorganism belonging to the archiascomycete fungus that comprises only three chromosomes, is characterized by a relatively quick division cycle. The generation time of the fission yeast under optimal growing conditions with all required nutritional support and appropriate temperature (30 °C) is approximately 3 h [12]. Therefore, to determine the acute effect of Cd exposure on the fission yeast, the cells were treated with the toxicant for 3 h, and longer incubation periods, 6 and 9 h of treatment, were performed for long-term effect determination. As cell survival is dramatically affected by Cd exposure, several working groups investigated cell responses to various Cd concentrations [16,17,18,19]. The IC_50_ value represents the metal concentration that inhibits cell growth to 50%. We exposed *S. pombe* cells to serially diluted Cd concentrations and determined that the IC_50_ value accounts for 51.68 μM of CdCl_2_ (Figure 1a). Under the experimental conditions, the growth ability of *S. pombe* cells over time decreased with increasing Cd concentrations, resulting in almost absolute cell growth abolishment after cell incubation with concentrations as high as 400 μM (Figure 1b). One possible reason for the altered cell growth is the defective chromosome segregation during mitosis. Indeed, increasing Cd concentrations caused an enhanced incidence of sister chromatid non-disjunction in a dose-dependent manner (Figure 1c).

### 2.2. Tor1 Deficiency Causes Higher Tolerance of Cells to Cd

**Figure 2:**
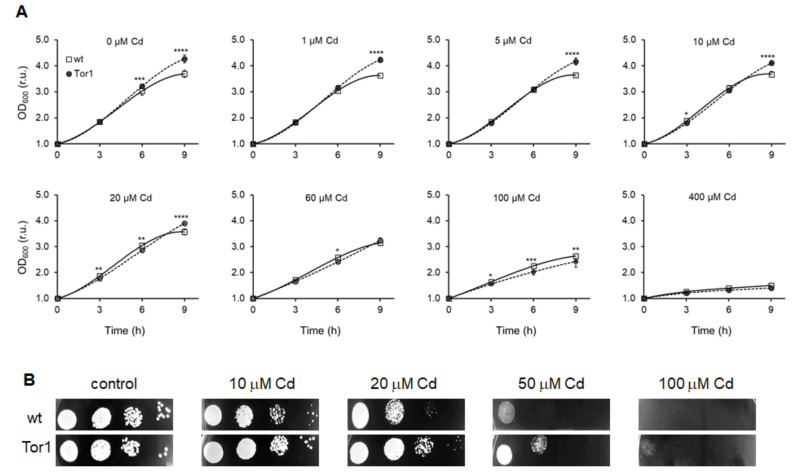
Growth of wild-type and Tor1-null cells exposed to Cd. (**A**) Growth curves of both tested yeast strains 3, 6, and 9 h after treatment with increasing Cd concentrations demonstrate the dose-dependent negative effect of increasing the Cd concentration on the cell growth (relative units, r.u. represent relative increase in OD_600_ compared to time point 0 h). Significant differences were detected among the growth rates of Tor1-depleted (full circles, Tor1) and wild-type (empty squares, wt) cells. Statistical analysis was determined by Duncan´s post-hoc test; each point indicates mean ± standard deviation (SD) (*n* = 8) and statistical significance is expressed with an asterisk (*) and set up as *p* < 0.05 *, 0.01 **, 0.001 ***. (**B**) The growth ability on solid YES (yeast extract plus supplements) plates of four times-diluted Tor1 and WT cell lines is compared. Where indicated, the cells are supplemented with the appertaining Cd concentration. Depicted are the representative pictures of four individual experiments.

To investigate the role of the TORC2 regulatory subunit Tor1 in Cd-mediated stress, wild-type and Tor1-depleted cells were exposed to different Cd concentrations and growth ability of the two respective strains was compared. Importantly, cells were cultured in rich YES (yeast extract with supplements) medium for 24 h at 30 °C and vigorous aeration, then diluted and again cultured for another 24 h under the same conditions before Cd supplementation, as the Tor1-null cells required a longer time to recover from the 4 °C storage. Strikingly, Tor1-deficient cells showed a substantially better growth ability compared to wild-type cells until the Cd supplementation reached a high concentration of up to 100 μM. Such a high concentration caused marked growth alterations in both strains, however with a quicker manifestation in the Tor1-null strain. An even higher concentration, 400 μM, abolished the cell growth of both strains (Figure 2a) (Supplementary Figure S1). Moreover, statistical analyses of variance (two-way ANOVA) revealed that the observed significant differences depend on both the Cd impact and the genotype as well as the interaction of the two conditions except for the 3 h of incubation with Cd (Supplementary Table S1/Growth). Consistent with previous findings representing cell growth in a liquid medium, spot test analyses on solid media revealed that the Tor1-deficient cells showed a higher tolerance towards Cd-induced stress. Cells depleted in Tor1 were able to form more dense spots on Cd-containing plates compared to wild-type cells (Figure 2b).

**Figure 3:**
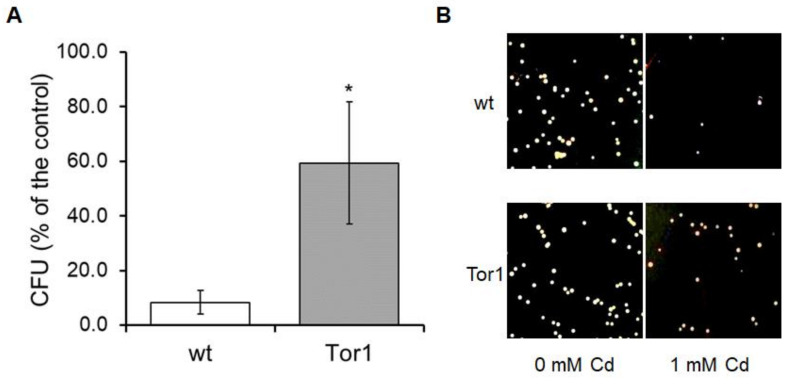
Colony formation of wild-type (wt) and Tor1-null (Tor1) cell lines in the presence of Cd. (**A**) Acute Cd contamination represented by cell exposure to high Cd concentrations (1 mM) for 3 h largely reduces the cell growth recovery. Ability to form new colonies on fresh YES (yeast extract plus supplements) plates was significantly lower in wild-type (wt) compared to Tor1-null (Tor1) cells. (**B**) Representative pictures of newly formed colonies from four independent experiments. Statistical significance expressed as *p* < 0.05 *, 0.01 **, 0.001 *** was determined by Student´s T- test; each bar indicates the mean ± standard deviation (SD) (*n* = 4).

The further determination of cell responses to Cd contamination expressed as colony forming unit (CFU) generation, representing the ability of cells to recover from acute Cd treatment, again confirmed the higher resistance of cells lacking Tor1 against Cd-mediated stress (Figure 3).

### 2.3. Tor1-Null Cells Show Higher Resistance Against Cd-Mediated Oxidative Stress

**Figure 4:**
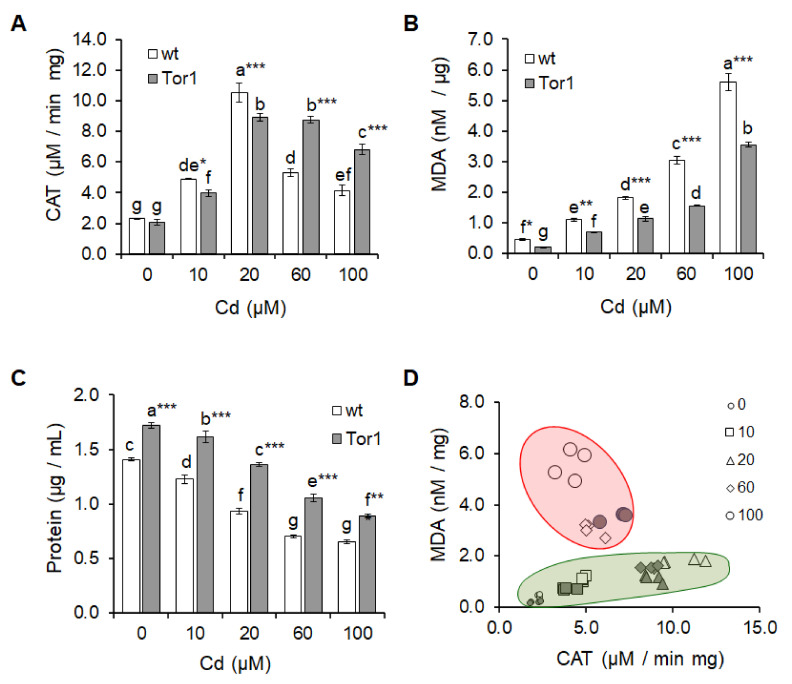
Cd-mediated oxidative stress is noticeably reduced in Tor1-null cells. Bars represent the mean values ± SD (*n* = 8) of catalase (CAT) activity (**A**), malondialdehyde (MDA) content (**B**), and protein amounts (μg/mL) (**C**) of wild-type (wt, white bars) and Tor1-deficient (Tor1, grey bars) cells supplemented with different CdCl_2_ concentrations for 3 h. Statistical significance is represented by asterisks (*). Number of * represents statistical significance and is determined as *p* < 0.05 *, 0.01 **, 0.001 ***; the same letters above bars indicate that these mean values are not significantly different. (**D**) Graphical illustration demonstrates the degree of Cd toxicity in correlation with the protective capacity of the two tested strains. The green circle depicts the “protective zone”, while the red circle depicts the “zone of toxicity”.

Cadmium indirectly elevates ROS formation, thereby subjecting cells to oxidative stress [20,21]. To determine the responses of wild-type and Tor1-deficient cells to Cd-mediated oxidative stress, we examined the catalase activity of both tested strains exposed to Cd. Interestingly, the catalase activity upon cell treatment with lower Cd concentrations (10 and 20 μM) was significantly higher in wild-type compared to Tor1-deficient cells. The lower catalase activity in Tor1-null cells suggests that these cells produce less ROS and thus do not require a high catalase activity. In comparison, wild-type cells were much more strongly affected already by lower Cd concentrations (Figure 4a). Higher Cd doses (60 and 100 μM), however, led to a dramatic increase in the catalase activity in cells lacking Tor1, thereby leading to their substantial protection. The Cd-mediated alteration of the antioxidant defense mechanism markedly decreased the catalase activity of wild-type cells, affecting their growth, which resulted in a noticeable drop in the protein level compared to Tor1-deficient cells (Figure 4A(a,c)). Another typical sign of oxidative stress is the lipid peroxidation characterized by enhanced malondialdehyde (MDA) formation as its end-product. The detection of MDA content revealed that the Tor1-null cells displayed significantly higher protection against lipid peroxidation compared to wild-type cells (Figure 4b). The catalase/MDA ratio displayed in Figure 4d represents two zones of Cd action and the protective capacity of the two tested strains. The green circle depicts the “protective zone”, while the red circle depicts the “zone of toxicity”. The picture shows that Tor1-null cells persist in the “protective zone” to higher Cd concentrations compared to wild-type cells. Hence, we suspect that Tor1 negatively regulates the defense mechanisms required for cell protection against the Cd-mediated alterations resulting from elevated oxidative stress. The two-way ANOVA statistical analyses revealed that the experimental specifications of Cd treatment, the genotype, and the interaction of the two conditions are responsible for the detected statistical differences (Supplementary Table S1/Biochemistry).

### 2.4. Effect of Cadmium Treatment on Wild-Type and Tor1-Null Cell Morphology

**Figure 5:**
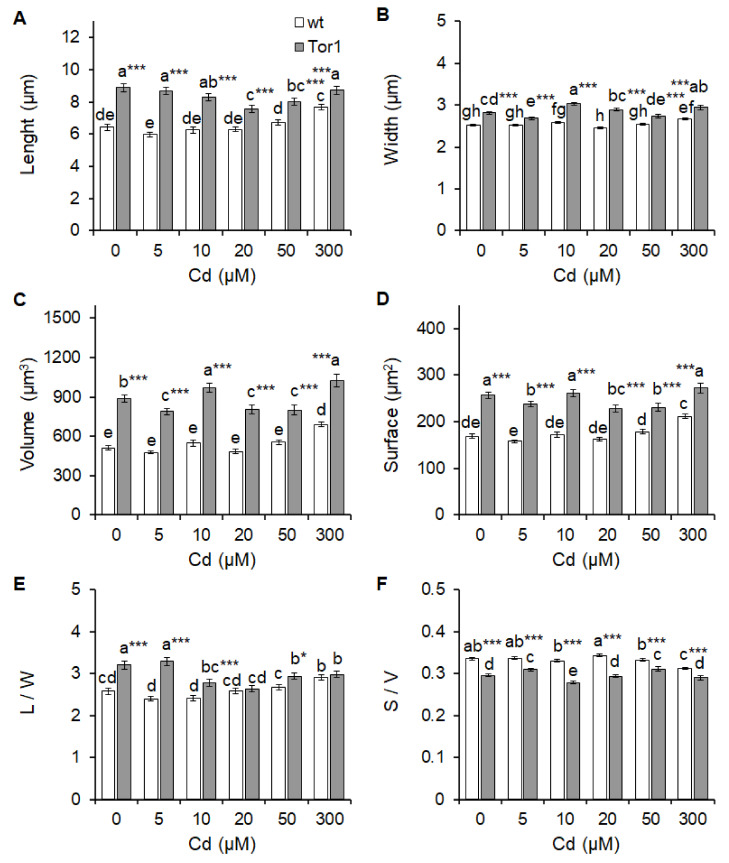
Morphological parameters of the Tor1-depleted and wild-type cells subjected to Cd. Determination of the cell length (**A**) and width (**B**) in μm of wild-type (wt, white bars) and Tor1-deficient (Tor1, grey bars) cells before and after Cd exposure. Calculation of the cell volume (**C**) in μm^3^ and surface (**D**) in μm^2^ of wt and Tor1 cells represents more objective analyses of shape changes between the wt and Tor1 cells upon Cd exposition. (**E**) Aspect ratio (lenght/width L/W) demonstrates changes in the cell shape and (**F**) the surface/volume (S/V) ratio represents changes in the size of tested cells. Statistical significance is determined by Duncan´s post-hoc test, defined with an asterisk (*) given as *p* < 0.05 *, 0.01 **, 0.001 ***; bars indicate mean ±SD (*n* = 100), and bars with identical letters are not significantly different.

Fission yeast is a unicellular self-living organism with a characteristic cylindrical rod shape 6–7 μm long and 2–3 μm wide. Cell assembly is an important parameter, as it is specific for every cell type and its organizational adjustment depends on the cell cycle phase. Exponentially growing cells double their size during mitosis by axial extension and divide into two equally long daughter cells by medial fission [22,23]. Challenging environmental conditions cause changes in the *S. pombe* morphology, indicating cellular transformations. The lengths and widths of both tested yeast strains were measured and compared. Figure 5 depicts the differences in the cell morphology parameters of the Tor1-depleted cells; the lengths and widths of 100 analyzed cells are greater compared to wild-type cells even under normal environmental conditions (Figure 5a,b). A drop in the cell length of Tor1-null cells was observed after the addition of 20 and 50 μM of cadmium, while the length of wild-type cells was significantly altered upon exposure to 300 μM of cadmium compared to the untreated control (Figure 5a). Cell width measurements revealed that Tor1-deficient cells respond more sensitively to cadmium exposure. resulting in significant differences in cell thickness upon treatment with 5, 10, and 300 μM of Cd, while wild-type cells show significant differences only after exposure to 300 μM of Cd compared to the untreated control (Figure 5b). Cell volume (μm^3^) and cell surface (μm^2^) analyses confirmed higher cell shape variations upon Cd treatment in Tor1-deficient cells than in wild-type cells. Changes in the mass gain (μm^3^) of Tor1-null cells were detected after treatment with all Cd concentrations. but only after the 300 μM of Cd treatment of wild-type cells compared to the untreated control (Figure 5c). The surface area (μm^2^) changed significantly under 5, 20, and 50 μM of Cd treatment of Tor1-null cells, and 300 μM Cd treatment of wild-type cells (Figure 5D). Shape modifications represented via aspect ratio were calculated as the length/width relation (L/W). Higher aspect ratio value indicates more cylindrical shape, while lower value indicates more spherical shape of the cell. Wild-type cells treated with Cd changed their shape significantly to be even more cylindrical after only 300 μM of Cd exposure, whereas Tor1-depleted cells displayed significant differences in the aspect ratio value, revealing a more spherical shape upon treatment with all Cd concentrations except for 5 μM (Figure 5e). The size of the cell is determined as the surface/volume (S/V) ratio; the lower the ratio, the bigger the cell. Significant differences in cell size upon Cd exposure were detected in both tested strains. Slight differences—however, reaching significance—were detected in wild-type yeast cells exposed to 10 or 50 μM of Cd, while more pronounced increases in cell size were detected upon 300 μM of Cd treatment. A more prominent cell size variability was exhibited in Tor1-deficient cells showing a significant increase followed by decrease in the cell size upon Cd treatment (Figure 5f). As the length and width of *S. pombe* cells is very uniform, which was evidenced by the very low standard deviations upon the measurement and evaluation of at least 100 cells per strain and experimental condition, it consequently resulted in a situation where even small differences among the tested strains and conditions reached statistical significance. Thus, the calculation of the cell surface, volume, their ratio, and the aspect ratio help to determine virtual differences among strains and environmental conditions in a more objective way. Hence, we believe that cadmium-induced cell morphology alterations differently affect wild-type compared to Tor1-null cells, suggesting the involvement of different regulatory pathways attempting to maintain size and shape under the control of the two tested strains. We therefore suspect that the Tor1-mediated signaling may be relevant for actin organization under Cd contamination in fission yeast. In accordance, the two-way ANOVA test of variables confirmed that the determined statistical differences in experimental conditions depend not only on Cd supplementation but also on the genotype. The interaction of the genotype and Cd treatment is shown to be significant for all of the observed differences except for volume, as it tightly did not reach statistical significance (Supplementary Table S1/Morphology). Representative pictures of the two analyzed strains subjected to the analyzed Cd concentrations are depicted in Supplementary Figure S2.

### 2.5. Ionome Disarrangement of Wild-Type and Tor1-Deficient Cells Exposed to Cd

**Figure 6:**
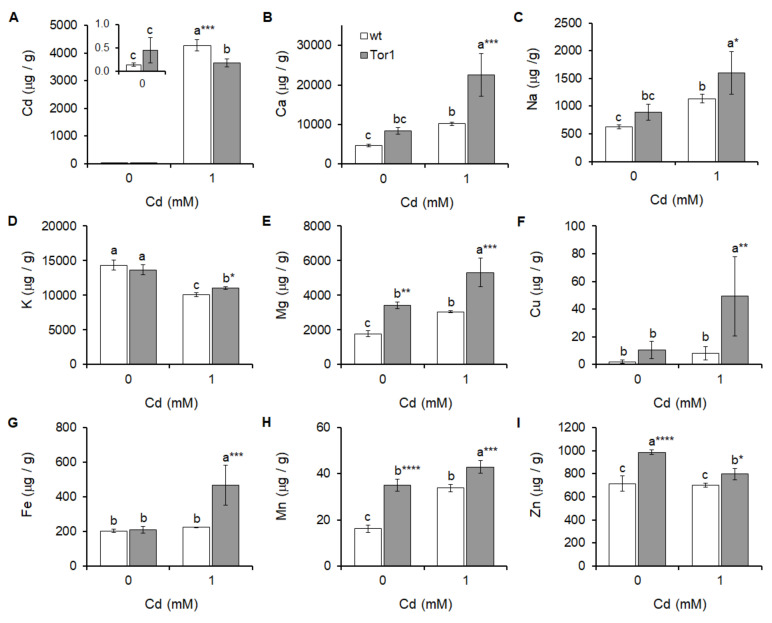
Ion content of wild-type (wt, white bars) and Tor1-deficient (Tor1, grey bars) cells before and after the Cd treatment. Determination of the mineral particles content by inductively coupled plasma – optical emission spectroscopy (ICP-OES) demonstrates differences in the (**A**) cadmium, (**B**) calcium, (**C**) sodium, (**D**) potassium, (**E**) magnesium, (**F**) copper, (**G**) iron, (**H**) manganese, and (**I**) zinc amounts of untreated or Cd (1 mM)-treated wt and Tor1 cells. Statistical significance determined by Duncan´s post-hoc test is expressed with an asterisk (*) and set up as *p* < 0.05 *, 0.01 **, 0.001 ***; identical letters above bars indicate no significant differences between the two values, white bars represent the mean ± SD of wild-type cells, grey bars represent the mean ± SD of Tor1-null cells (*n* = 4).

The cell ionome represents the elemental composition of the cell. A balanced micro and macro element content is required for normal cell physiology. Analyses of the yeast ion content by the ICP-OES detection system of the two tested strains revealed that the Tor1-depleted cells displayed significant differences in their Mg^2+^, Mn^2+^, and Zn^2+^ contents compared to wild-type cells, while the content of other studied elements did not show significant differences. The acute environmental contamination caused by 1 mM of Cd led to massive changes in mineral element composition. Wild-type cells displayed significantly increased Ca^2+^, Na^+^, Mg^2+^, and Mn^2+^ levels and decreased K^+^ levels, while no significant difference was detected in the Cu^2+^, Fe^3+^, and Zn^2+^ levels (Figure 6b–i white bars). In contrast, in Tor1-deficient cells the content of all analyzed elements changed. Cd treatment enhanced the levels of Ca^2+^, Na^+^, Mg^2+^, Cu^2+^, Fe^3+^, and Mn^2+^, whereas the K^+^ and Zn^2+^ level decreased (Figure 6b–i grey bars). Interestingly, the cellular Cd incorporation was significantly lower in Tor1-null cells compared to wild-type cells (Figure 6a).

Additional statistical analyses by the two-way ANOVA show that the ionome balance of all the analyzed elements is altered due to both the Cd treatment and the genotype, except for the difference in the K^+^ amount, which is not genotype-dependent. The interaction of the two experimental conditions (G x Cd) appeared not to be responsible for the differences detected in the Na^+^, Mg^2+^, and Cu^2+^ levels (Supplementary Table S1/Ionome).

**Figure 7:**
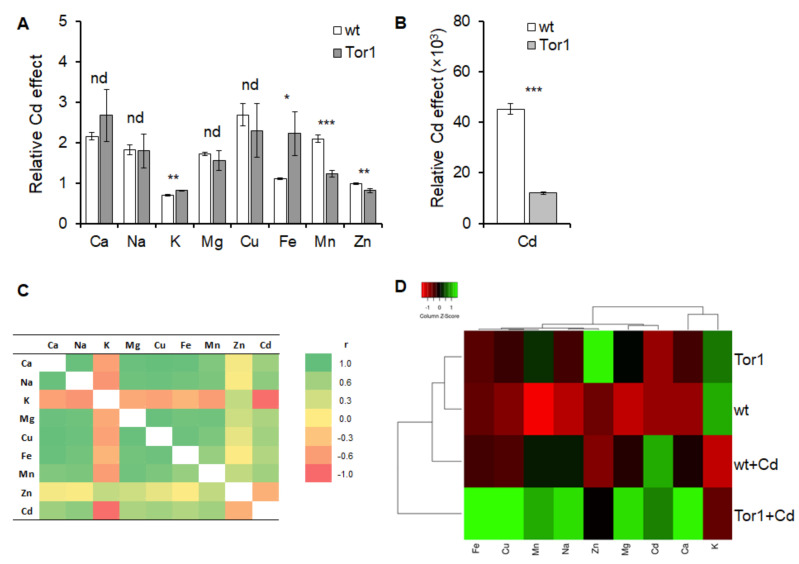
The effect of Cd supplementation on wild-type and Tor1-null cells ionome. (**A**) Changes in the mineral elements content after Cd treatment expressed as ratio of the respective untreated strain shows that the increase in Ca^2+^, Na^+^, Mg^2+^, and Cu^2+^ levels is comparable among the two tested strains, whereas the increase in Mn^2+^ and Zn^2+^ and in the wild-type (wt, white bar) strain is significantly higher compared to the Tor1-deficient (Tor1, grey bar) cells, while the decrease in the K^+^ levels and the increase in the Fe^3+^ amounts are significantly higher in the Tor1 strain. (**B**) Cd content in WT and Tor1 cells expressed as the ratio of the respective untreated strain. Statistical significance is specified by Duncan´s post-hoc test, expressed by asterisks (*) and shown as *p* < 0.05 *, 0.01 **, 0.001 *** nd above the bar indicates that the two mean values are not significantly different; individual bar represents the mean ± SD (*n* = 4). (**C**) Correlation analysis showing mutual correlations of tested mineral elements, ranging from a highly positive Pearson´s correlation coefficient (r = 1) to a highly negative correlation coefficient (r = −1). (**D**) Data-driven heatmap expresses a range of values which show mutual correlations of data clusters established from each tested condition and the ion content in relation to the control.

To determine the virtual Cd effect on changes in the ionome, the content of each studied mineral element was subtracted from the original ion content of the respective untreated control. Strikingly, statistically significant differences were detected only in the amounts of Mn^2+^ and Zn^2+^, which were lower in Tor1-null cells, and the K^+^ and Fe^3+^, which reached higher concentrations in cells lacking Tor1 compared to wild-type cells (Figure 7a). In line with the initial observation, the relative Cd uptake by the Tor1-deficient cells was markedly reduced compared to wild-type cells (Figure 7b), suggesting the role of Tor1 kinase in metal trafficking. Pearson´s correlation analysis expressing positive or negative Z-scores of mutual relations among the studied ions demonstrates a strong positive correlation among all the tested elements, except for K^+^ and Zn^2+^, which show negative or no correlation, respectively (Figure 7c). A data-driven heatmap expressing a range of values shows a reciprocal cluster correlation between each tested condition and the ion content in relation to the control (Figure 7d). Ion migration sustains the mineral element balance and serves as a support machinery to maintain the internal cell homeostasis. The cadmium uptake by the Tor1-deficient cells is considerably lower compared to the wild-type cells, which might contribute to the growth ability protection of these cells and, moreover, suggests the role of the *S. pombe* Tor1 in ion transport mechanisms.

## 3. Discussion

Cadmium toxicity is a subject of serious concern, as it seamlessly accumulates in soft tissues such as the kidneys or liver with a low remediability rate of the body and is characterized by a considerably long degradation time in biological tissues (20–30 years in humans) [24,25]. As Cd is classified as carcinogenic to humans through multifactorial mechanisms including ROS enhancement in turn leading to genome instability [20,26,27], we investigated the effect of Cd on the fundamental processes of all living organisms, cell growth, cell morphology, or homeostasis. For the analyses, we used the fission yeast *S. pombe* as a prominent model organism in the field of eukaryotic molecular and cellular biology. As the fission yeast shares many molecular, genetic, and biochemical properties with higher eukaryotes, it is often referred to as a “micro-mammal”. Studies of *S. pombe* have led to the discovery of genes involved in the regulation of fundamental processes common for yeast and higher species that, among others, include mRNA splicing, post-translational modifications such as protein N-glycosylation, DNA replication, cell-cycle regulation, or nutrient-sensing pathways such as the TOR network [28,29,30]. The TOR kinase has been described to play a critical role in nutritional signaling not only in fission yeast but also in other eukaryotic organisms [7,31]; we therefore investigated responses of the Tor1-deficient *S. pombe* cells to Cd treatment. As the cell proliferation exquisitely depends on nutrient availability, our studies were performed with the use of rich YES media providing every nutritional requirement for the cell with the aim to avoid concomitant starvation and heavy metal-derived phenotypes. A rich nutrient supply stimulates a high level of protein synthesis, thus promoting division. Cell cycle progression under specific environmental context coordinates TOR kinase by the regulation of complex processes in the cell that include metabolism, migration, or division [32]. Despite ample nutrient availability, the increasing Cd concentration in the growth media led to severe growth impairments of cells which might be associated with the Cd-mediated impairment of protein folding in the endoplasmic reticulum, resulting in the unfolded protein response (UPR). In vivo studies revealed that the deleterious effect of Cd is associated with the Cd-induced misfolding and aggregation of nascent proteins undergoing the process of synthesis or folding [16,33,34]. Although the TOR activity is tightly related to the environmental conditions of the cell, cells depleted in the Tor1 kinase showed growth retardation with noticeably slower gradation compared to wild-type cells (Figure 2 and Figure 3). Whether the protective phenotype of mutated cells is connected to the yet-unknown Tor1 negative regulation of Cd-derived protein misfolding is a topic for further investigation. Additionally, our data clearly, although surprisingly, demonstrate that the Tor1 deficiency significantly protects cells against Cd-induced oxidative stress (Figure 4). The redox-inactive Cd was reported to indirectly accelerate the production of reactive oxygen species (ROS) ^·^O_2_^−^ and H_2_O_2_ in yeasts in a dose-dependent manner, resulting in the activation of the antioxidant enzymes superoxide dismutase (SOD) and catalase (CAT) [35,36]. In our study, no difference was observed in the catalase activity of both tested yeast strains before Cd treatment. Growth media supplementation with lower cadmium concentrations (10 and 20 μM) caused an acceleration in the CAT activity of the analyzed yeast strains; however, for the Tor1-deficient cells a weaker CAT activity was sufficient to eliminate the negative effect of Cd. Higher Cd concentrations (60 and 100 μM) attenuated the CAT activity of wild-type cells, pulling cells into oxidative stress, while the Tor1-null cells were able to keep a high CAT activity, thereby preserving cells from stress. As the catalase activity enhancement and/or inhibition largely varies depending on the metal dose and time of its exposure [37,38,39], the determination of the malondialdehyde (MDA) content representing the end-product of lipid peroxidation serves as an oxidative stress indicator. Strikingly, the content of MDA was significantly lower in cells depleted in Tor1, indicating that the abolishment of the Tor1 activity might protect cells from Cd-mediated oxidative stress. A similar alleviation of oxidative stress caused by Cd exposure was determined in *Saccharomyces cerevisiae* cells overexpressing the OLE1 gene, most probably due to protection of the cytoplasmic membrane from damage [40]; however, the exact mechanism needs to be elucidated. As another indicator of the cell condition is its shape, we have determined the morphometric parameters of the two analyzed strains upon Cd treatment. *S. pombe* is a unicellular microorganism with a cylindrical rod shape, typically 6–7 μm in length and 2–3 μm in width. Cells grow by axial extension to the opposite poles; after doubling their original size, they enter mitosis and divide by medial fission into two identical daughter cells. Cell exposure to a challenging environment triggers signaling through the network regulatory motifs, so the shape of cells is often the result of the prior signaling. It reflects changes in the cell surrounding conditions resulting in alterations in the cytoskeleton organization and dynamics. The complex physico-chemical properties of the regulatory network enables the cell to dynamically control information processing as signals flow across the cell surface and respond with adequate shape modifications [23,41,42]. Cd-dependent alterations in the cytoskeletal dynamics caused changes in the cell morphology in regard to the used concentration, similarly as was previously reported for other different types of cells [43,44,45]. Generally, lower Cd doses led to slight decreases in the cell size, while higher concentrations were manifested by the generation of large irregularly shaped cells. As shown by others, cells depleted in Tor1 are bigger compared to wild-type cells [46]. Upon Cd exposure, Tor1-depleted cells remained bigger; however, the changes in their shape did not correspond to the shape modifications of wild-type cells, suggesting the role of Tor1 signaling in cell morphology determination. As the cell shape sustainment is a highly complex system regulated by a large variety of signaling events, including the key morphology regulator Rho GTPase Cdc42 [47,48,49,50,51], it would be very interesting to investigate the precise function of the Tor1 signaling in these processes. In addition to morphological alterations, Cd, through the interaction with receptors and ion channels on the cell surface, interrupts the ion balance required for the cell homeostasis maintenance. The determination of the cellular quotient of ions revealed the shift in the range of mineral element concentrations upon Cd exposure of wild-type and Tor1-null cells. The acute Cd-evoked stress induced by cell treatment with a high Cd concentration (1 mM) elicited a significant boost in the intracellular Ca and Mg ions of both tested strains, thereby counteracting the Cd toxicity. This is in agreement with previous reports showing that Cd exposure affects Ca homeostasis, increases cytosolic Ca levels, and abuses Ca or Mg channels for self-transportation; moreover, the cell defense efficiency largely depends on the availability of extracellular Ca [52,53,54,55]. The data showing changes in the amounts of almost all the tested elements are consistent with the evidence that cadmium alters the ion homeostasis on different levels as it interferes with vast molecules of the major cellular regulatory pathways not only in yeast but also in higher organisms [4,56,57]. Signaling through multiprotein complexes that contain the serine/threonine kinase TOR, such as the TORC2 complex with an array of substrates, controls a range of cellular functions. It is therefore not surprising that the fission yeast TORC2 catalytic subunit Tor1 is involved in processes directed to regulate ion trafficking. However, to our surprise the Cd uptake by Tor1-deficient cells is considerably lower compared to wild-type cells. However, this proposition might contribute to the overall protective phenotype of Tor1 mutated cells. Overall, our findings here establish the role for Tor1 kinase in the cell survival processes in a Cd-rich environment.

## 4. Materials and Methods

### 4.1. Yeast Strains, Media and Growth Conditions

To determine the response of *Schizosaccharomyces pombe* cells to Cd-induced stress, wild-type *SP72 h+ ade6-M210 ura4-D18 leu 1-32* and the Tor1 kinase-deficient strain *SP761 h+ ade6-M210 ura4-D18 leu 1-32 tor1::KanMX* were used for the analyses. For the double control of acquired results, a Tor1-deficient strain from the Bioneer deletion collection (V3-P11-54) kindly provided from Dr. Gregan was used for comparative experiments showing identical phenotypes (data not shown). Based on the protein fingerprint by matrix-assisted laser desorption/ionization time-of-flight mass spectrometry (MALDI-TOF MS Biotyper; Bruker Daltonics, Bremen, Germany), the used cultures of both analyzed yeast strains were validated as pure *S. pombe* cultures (Supplementary Figure S1). Cells from the 1 mL fresh overnight culture were used for the ethanol-formic acid extraction, covered with 1 µL of matrix solution (saturated solution of α-cyano-4-hydroxycinnamic acid in 50% acetonitrile with 2.5% trifluoroacetic acid; Bruker Daltonics), and subsequently dried in air for 15 min. Protein spectrum of the sample was generated by the Biotyper software, version 3.0 (Bruker Daltonics) and analyzed as previously described [58].

Yeasts were cultured in a standard rich YES (YE = yeast extract, S = adenine, L-histidine, L-leucine, L-lysine and uracil, AppliChem GmbH, Darmstadt, Germany) medium prepared according Forsburg and Rhind [59]. Cadmium (CdCl_2_, Sigma-Aldrich, St. Louis, MO, USA) in an appropriate concentration was added where indicated. To prepare the solid media, 20 g/L of agar was added to the liquid culture media. Optimal growth conditions were ensured by cell incubation at 30 °C and proper aeration by vigorous shaking (150 rpm).

### 4.2. IC_50_ Value Determination

The cadmium concentration leading to a 50% cell growth reduction represents the IC_50_ value. Exponentially growing wild-type *S. pombe* cells were exposed for 6 h at 30 °C to decreasing Cd concentrations using the log2 dilution from 400 µM to 1.6 µM and, as a control, medium without Cd supplementation was used. The effect of Cd on cell growth was determined spectrophotometrically as the difference in light absorbance at 600 nm (OD_600_) by the Glomax Multi Detection System spectrophotometer (Promega Corporation, Madison, WI, USA) before and after 6 h of incubation with Cd. The difference in the cell density causes a change in the light absorbance that determines Cd-mediated alterations in the growth ability. The cell density ratio representing the cell growth was calculated from OD_600_ values before and 6 h after Cd supplementation and the IC_50_ value was determined by the use of an IC_50_ calculator [60].

### 4.3. Growth Rate

The overnight culture of wild-type and Tor1-deficient cells was diluted to OD_600_ = 0.3 and cultured for another 24 h at 30 °C and 150 rpm shaking conditions because Tor1-null cells are perceptibly more cold-sensitive and thus require a longer time to recover from 4 °C storage. Next, after the dilution of the cell culture to OD_600_ = 0.3, the effect of Cd supplementation (1, 5, 10, 20, 60, 100, 400 μM) on the cell growth was determined. The control group was left untreated. Cells were incubated for 3, 6, and 9 h in 24-well plates at 30 °C under 150 rpm shaking conditions, and the OD_600_ was measured at each indicated time point. The ratio of the cell density increase at every time point compared to the control time point 0 h represents the growth rate.

### 4.4. Immunostaining and Microscopy

The segregation of chromosomes under Cd-mediated stress conditions was analyzed with the use of immunostaining followed by fluorescence microscopy, as was previously described by Rabitsch et al. [61]. Shortly, a yeast strain with GFP-labeled chromosome II (JG 15,457 *cen2(D107):KanR-ura4+-lacO his7+::lacI-GFP*) in which a chromosome locus close to the centromere is visualized through a specific binding of the LacI-GFP fusion repressor to the lacO tandem repeats sequence was grown in YES medium to OD_600_ = 1. Cells were incubated with 0, 50, and 100 μM of Cd for 3 h; fixed with 4% paraformaldehyde (PFA) in PEMS (PEM + 1.2 M sorbitol); and stained with primary TAT1 mouse monoclonal anti-tubulin (1:200) and rabbit polyclonal anti-GFP (Thermo Fisher Scientific; Waltham, USA, 1:400) antibodies. After 16–24 h on a wheel at RT and 3 washing steps with PEMBAL, the samples were resuspended in PEMBAL (PEM + 1% BSA + 0.1% NaN_3_) containing anti-mouse-Alexa546 and anti-rabbit-Alexa488 (Thermo Fisher Scientific; Waltham, USA) both diluted 1:500 and incubated as before. After washing, the samples were mounted on poly-L-lysine-coated cover slips in Vectashield with DAPI (Vector Laboratories, Burlingame, CA, USA) to visualize the DNA. Analyses were performed on an inverted fluorescent microscope equipped with a digital camera (Leica DMI 6000, Leica microsystems, Wetzlar, Germany).

### 4.5. Analyses of the Cell Growth Restoration after Cd Treatment

The cell quantity from the overnight culture of both tested strains was adjusted to 1 × 10^6^ cells/mL, and 0, 50, 100, 300, and 1000 μM of Cd was subjected to the liquid growth medium. Cells were incubated for 3 h at 30 °C under vigorous shaking, diluted 1:1000, and 10 μL of the respective strain treated or untreated with Cd were uniformly spread on solid YES medium and cultured at 30 °C for two days until colonies were formed. Colonies were counted and colony forming units (CFUs) of cells from the heavy metal-containing cultures were calculated as a percent of the formed colonies from untreated cultures, representing the cell regeneration ability after the acute Cd (1 mM) treatment.

### 4.6. Spot Test Analyses

Solid YES media were enriched by 0, 10, 20, 50, and 100 μM of CdCl_2_. Serially diluted wild-type and *tor1*-null cells from the overnight culture were placed on plates, resulting approximately in 10,000, 1000, 100 and 10 cells/spot. After 2–3 days of incubation at 30 °C spots appeared, and their size and density have been compared.

### 4.7. Biochemical Analysis

For biochemical analysis, the yeast cells were incubated with the indicated Cd concentrations for 3 h at 30 °C followed by centrifugation (60 s, 8500 rpm), washed 3 times with sterile H_2_O, and finally resuspended in PBS buffer (pH 7.0). Cells were stored until further analysis at −80 °C. Prior to analysis, the samples were homogenized by sonication (Digital Sonifier 450, Branson Ultrasonics Corp, Danbury, CT, USA) 3× at 30 s intervals on ice. Cell debris was removed by 15 min of centrifugation at 14,000 rpm at 4 °C. The supernatant was collected and the protein levels, catalase activity, and MDA content were determined.

Catalase (CAT) activity was assayed according to Aebi (1984) [62]; briefly, the H_2_O_2_ decomposition was measured as the decrease in absorbance at 240 nm for 90 sec at the Agilent Cary 60 UV/VIS spectrophotometer (Agilent Technologies, Santa Clara, CA, USA). The reaction was initialized by the addition of 2.5 mM of H_2_O_2_ to the respective sample, and the catalase activity was calculated with the use of a specific molar absorption coefficient at 36 mM^−1^ cm^−1^.

The determination of lipid peroxidation through the malondialdehyde (MDA) content measurement was performed according to Pekmez et al. [63] using the thiobarbituric acid (TBA) method with small modifications according to Garre et al. [64]. After sonication on ice, 300 µL of the supernatant from each sample was added to 600 µL of TBA solution (15% trichloroacetate TCA containing 0.375% (w/v) TBA) and subsequently incubated at 95 °C for 30 min. Afterwards, the sample was rapidly cooled on ice and centrifuged at 8500 rpm for 60 sec. The absorbance of the supernatant was measured at 532 and 600 nm at the Agilent Cary 60 UV/VIS spectrophotometer. The calculation of the MDA content with the use of the molar absorption coefficient at 153 mM^−1^ cm^−1^ resulted in the assessment of the MDA amounts, expressed as nmol·mg^−^^1^ of protein. The protein concentration was determined at 600 nm using the Bradford assay [65] with bovine serum albumin (Sigma-Aldrich, St. Louis, MO) as a standard. Four biological replicates were performed for biochemical analysis and the experiment was repeated twice.

### 4.8. Yeast Morphology Characterization

Exponentially growing wild-type and Tor1-depleted cells were incubated with different Cd concentrations for 3 h. Native microscope slides of the respective strain were prepared and imaged by bright-field microscopy with a 40× magnification on an inverted microscope (Leica DMI 6000, Leica microsystems, Wetzlar, Germany). The ImageJ software v. 1.52r (National Institutes of Health, USA) was availed for cell morphometric measurements.

Cell volume (*V*; µm^3^) was calculated as:

(1) $$V=\frac{4}{3}\pi LW^{2},$$

where *L* represents the cell length and *W* is the cell width.

Cell surface (*S*; µm^2^) was calculated as:

(2) $$S=2\pi \left(W^{2}+LW\frac{arcsin\varepsilon }{\varepsilon }\right),$$

where factor *ε* is calculated as:

(3) $$\varepsilon =\frac{\sqrt{L^{2}-W^{2}}}{L}$$

### 4.9. Pre-Analytical Sample Preparation of Yeast

Sample processing for the ion content analyses as described by Pozgajova et al. [66] involves the incubation of the examined yeast strains from the overnight culture with Cd (1 mM) for 3 h at 30 °C while the control is left untreated. Samples were washed 3x with deionized water, pelleted, and dried for 12 h at 50 °C. Desiccated pellets were then weighted and placed into PTFE digestion tubes containing 5 mL of extra pure HNO_3_. The full mineralization of each sample was achieved through pressure microwave digestion with the ETHOS-One (Milestone, Srl., Italy) system. Mineralized samples were filtered through quantitative Munktell filter paper No. 390 (Munktell and Filtrak, Bärenstein, Germany) into 50 mL volumetric flasks prior to the analysis. Each sample was prepared in 3 replicates.

### 4.10. Ionome Quantification

The ion content of the mineralized yeast samples in mg·kg^−^^1^ of dry matter was determined with the use of inductively coupled plasma optical emission spectroscopy ICP-OES 720 (Agilent Technologies Australia Pty Ltd.), with the detection limit for Cd as 0.05 μg/kg.

### 4.11. Statistical Analysis

Data are presented as the mean ± standard deviation (SD). The statistical significance of the detected differences was analyzed by an ANOVA and Duncan´s test using the Statistica 10 software (StatSoft Inc., Tulsa, OK, USA). Lavene´s and Cochran tests specified the data homogeneity and the normal distribution of experimental results. The correlation analysis of the individual ion content in the tested yeast strains was determined by the Pearson correlation coefficient (r_P_). The generation of *Z*-scores and clustering was performed with the use of Heatmap3. The limits of statistical significance were set up as *p* < 0.05 *, 0.01 **, 0.001 *** for all analyses.

## 5. Conclusions

Although Cd toxicity is associated with serious health threats including cancer in higher organisms, many questions concerning cell responses to Cd-mediated effects remain yet to be answered. Our results for the first time demonstrate the ability of cells depleted in Tor1 to better cope with Cd-mediated oxidative stress. The lipid peroxidation in Tor1-null cells is significantly lower compared to wild-type cells, and the catalase is able to protect Tor1-deficient cells at higher Cd concentrations. Moreover, Tor1 signaling is involved in the shape responses of cells exposed to Cd. Strikingly, Tor1 deficiency mitigates Cd accumulation in the cell, thereby hindering its negative impact. Hence, we show that the TORC2 catalytic subunit Tor1 is involved in cadmium-mediated cell impairment in fission yeast. The data presented here suggest the yet-undefined role of Tor1 signaling in Cd trafficking, however further investigations are required to uncover the exact mechanism of this action.

## Supplementary Materials

Supplementary materials can be found at https://www.mdpi.com/1422-0067/21/21/7847/s1. Supplementary Figure S1. Representative pictures of Tor1 depleted (Tor1) and wild type (wt) cells subjected to designated Cd concentrations. Supplementary Figure S2. Representative pictures of Tor1 depleted (Tor1) and wild type (wt) cells subjected to designated Cd concentrations. Supplementary Table S1. Statistical analyses by two-way ANOVA for changes in the cell growth, biochemistry, morphology and ionome. G—genotype, Cd—different Cd concentration, G × Cd—interaction of G and Cd effects.
