# Supplementary material for: Cadmium-Induced Cell Homeostasis Impairment is Suppressed by the Tor1 Deficiency in Fission Yeast
Source: Int J Mol Sci. 2020 Oct 22;21(21):7847. doi: 10.3390/ijms21217847 (PMC7660220; doi:10.3390/ijms21217847)
Supplement: Supplementary file 1 [file ijms-21-07847-s001.pdf]

Supplementary Tab 1 Statistical analyses by two-way ANOVA for changes in the cell growth, biochemistry, morphology and ionome. G – genotype, Cd – different Cd concentration, G×Cd – interaction of G and Cd effects.

	Trait	Effect	SS	MS	F	*p*
Growth	Growth 3h	G	0.1204	0.1204	41.80	0.000000
		Cd	4.5772	0.4577	158.89	0.000000
		G×Cd	0.0363	0.0036	1.26	0.269328
	Growth 6h	G	0.2164	0.2164	27.03	0.000002
		Cd	43.3435	4.3344	541.31	0.000000
		G×Cd	0.6000	0.0600	7.49	0.000000
	Growth 9h	G	0.5237	0.5237	44.36	0.000000
		Cd	91.0928	9.1093	771.63	0.000000
		G×Cd	2.7621	0.2762	23.40	0.000000
Morphology	L	G	956.28	956.28	245.07	0.000000
		Cd	183.78	36.76	9.42	0.000000
		G×Cd	123.56	24.71	6.33	0.000008
	W	G	26.803	26.803	254.97	0.000000
		Cd	7.589	1.518	14.44	0.000000
		G×Cd	3.238	0.648	6.16	0.000012
	V	G	33268171	33268171	424.598	0.000000
		Cd	7056753	1411351	18.013	0.000000
		G×Cd	852234	170447	2.175	0.054632
	S	G	1559860	1559860	405.77	0.000000
		Cd	283651	56730	14.76	0.000000
		G×Cd	57505	11501	2.99	0.010873
	L / W	G	42.168	42.168	73.17	0.000000
		Cd	20.502	4.100	7.11	0.000001
		G×Cd	26.428	5.286	9.17	0.000000
	S / V	G	0.3701	0.3701	278.27	0.000000
		Cd	0.0838	0.0168	12.61	0.000000
		G×Cd	0.0459	0.0092	6.90	0.000002
Biochemistry	Proteine	G	2.3624	2.3624	346.42	0.000000
		Cd	7.4237	1.8559	272.16	0.000000
		G×Cd	0.0939	0.0235	3.44	0.012618
	MDA	G	9.3486	9.3486	206.656	0.000000
		Cd	87.4327	21.8582	483.185	0.000000
		G×Cd	4.6615	1.1654	25.761	0.000000
	CAT	G	4.435	4.435	12.646	0.001272
		Cd	255.918	63.980	182.439	0.000000
		G×Cd	41.104	10.276	29.302	0.000000
Ionome	Cd	G	298951	298951	19.632	0.002195
		Cd	46916450	46916450	3080.989	0.000000
		G×Cd	299343	299343	19.658	0.002186
	Na	G	402285	402285	9.2605	0.015988
		Cd	1110969	1110969	25.5743	0.000981
		G×Cd	29062	29062	0.6690	0.437085
	K	G	7.725818E+04	7.725818E+04	0.280	0.611064
		Cd	3.580865E+07	3.580865E+07	129.779	0.000003
		G×Cd	2.101637E+06	2.101637E+06	7.617	0.024680

Continue supplementary Tab 1

	Trait	Effect	SS	MS	F	*p*
Ionome	Ca	G	1.913754E+08	1.913754E+08	25.1008	0.001040
		Cd	2.875370E+08	2.875370E+08	37.7134	0.000277
		G×Cd	5.583806E+07	5.583806E+07	7.3237	0.026814
	Mg	G	11529885	11529885	61.3339	0.000051
		Cd	7483269	7483269	39.8077	0.000230
		G×Cd	281153	281153	1.4956	0.256149
	Cu	G	13767.20	13767.20	21.0231	0.001790
		Cd	15794.10	15794.10	24.1182	0.001177
		G×Cd	1606.63	1606.63	2.4534	0.155905
	Fe	G	47307.7	47307.7	14.0022	0.005689
		Cd	58283.3	58283.3	17.2508	0.003194
		G×Cd	41686.8	41686.8	12.3385	0.007931
	Mn	G	589.61	589.61	122.274	0.000004
		Cd	491.95	491.95	102.023	0.000008
		G×Cd	70.74	70.74	14.670	0.005017
	Zn	G	101455	101455	53.739	0.000081
		Cd	30019	30019	15.901	0.004019
		G×Cd	22510	22510	11.923	0.008656

Note: SS – sum of square, MS – mean square, F – F ratio, *p* – probability, L – cell length, W – cell width, V – cell volume, S – cell surface

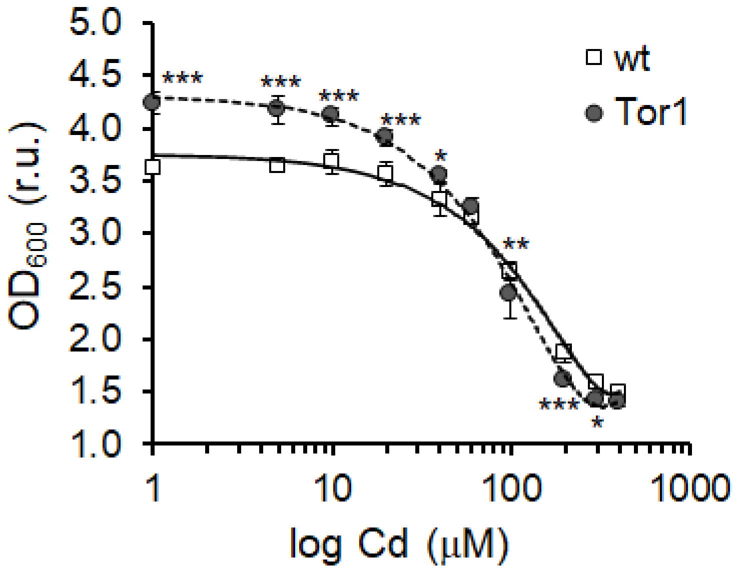

Supplementary Figure 1 Growth inhibition by increasing Cd concentrations of wt and Tor1 null cells. Optical density is determined at 600nm (OD_{600}) and represented by relative units (r.u.) of the total growth rate calculated as an increase in the cell density after 9 hours if incubation compared to the 0 h time point. Asterisks indicate statistical significance in slopes between the two curves.

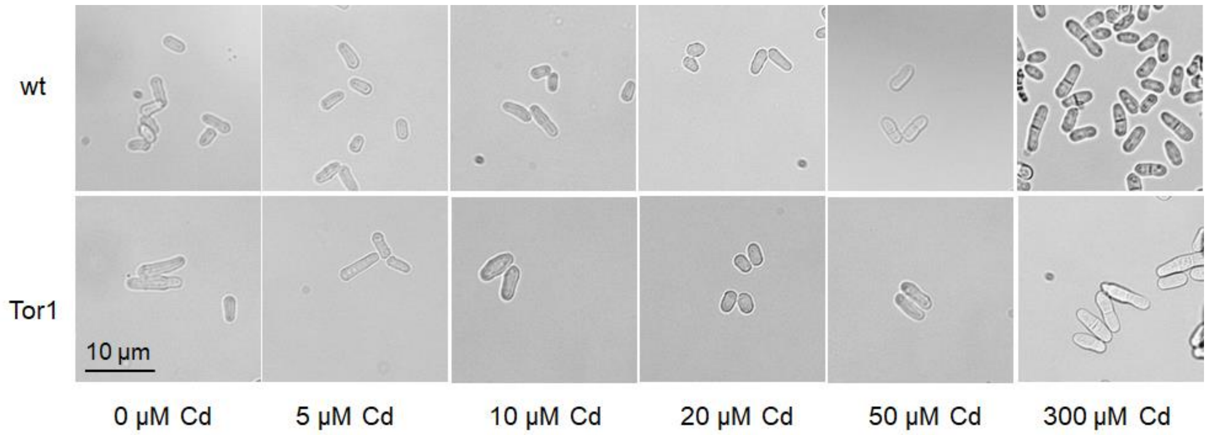

Supplementary Figure 2 Representative pictures of Tor1 depleted (Tor1) and wild type (wt) cells subjected to designated Cd concentrations
